# Cytomorphological features of Hürthle cell carcinoma: A report of two cases with review of literature

**DOI:** 10.4103/0970-9371.73303

**Published:** 2010-10

**Authors:** Kavita Mardi, Neelam Gupta, Sudarshan Sharma, Lalita Negi

**Affiliations:** Department of Pathology, Indira Gandhi Medical College, Shimla, Himachal Pradesh, India

**Keywords:** Hürthle cell carcinoma, Hürthle cell neoplasm, thyroid, fine needle aspiration cytology

## Abstract

The use of the term “Hürthle cell neoplasm” as the gold standard should be discouraged as it makes evaluating these lesions more confusing. Recently, a number of studies have been conducted to define criteria that are more specific for Hürthle cell carcinoma (HCC). We herein report two cases of HCC of thyroid which were accurately diagnosed preoperatively using various cytological features described in the recent studies. A review of the literature is also presented.

## Introduction

Accurate diagnosis of Hürthle cell neoplasms (HCNs) is very important. The frequent presence of Hürthle cells in nodules of various non-neoplastic thyroid lesions, particularly Hashimoto’s thyroiditis and multinodular goiter, makes cytological distinction of HCN from these non-neoplastic lesions difficult. We describe cytological features of two cases of Hürthle cell carcinomas (HCCS) and review the criteria used to distinguish HCC from other non-neoplastic and neoplastic Hürthle cell lesions.

## Case Report

We report two cases of HCC here. Both the cases were diagnosed as HCC in the pre-operative cytological evaluation and were confirmed on postoperative histopathological analysis.

### Case 1

**Figure 1:**
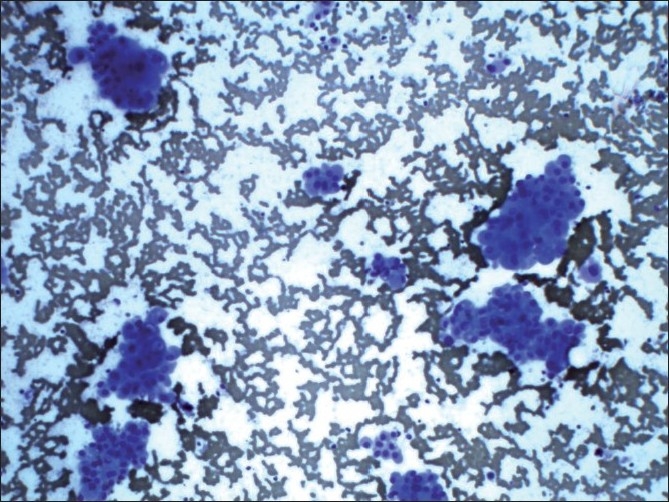
Monolayered sheets and overlapping clusters of Hürthle cell showing pleomorphism, abundant basophilic cytoplasm, eccentric to centrally placed round nucleus with variably prominent nucleoli (Giemsa, ×100)

A 57-year-old male presented with a rapidly enlarging mass in the neck for 5 months. He complained of dysphagia and difficulty in breathing. On examination, there was a nodular swelling involving both lobes of thyroid, measuring 15×10 cm. The lump was fixed to the underlying structures and computed tomography (CT) scan was suggestive of a malignant neoplasm. Fine needle aspiration (FNA) of the mass showed cellular smears comprising monomorphic population of Hürthle cells arranged in monolayered sheets, overlapping clusters, isolated cells along with some bare nuclei. The cells showed little pleomorphism, abundant basophilic cytoplasm, eccentric to centrally placed round nucleus with variably prominent nucleolus [Figure 1]. Colloid was not seen. Instead, background showed necrosis in some areas. Keeping in view the cytological findings, namely the cellularity, pleomorphic overlapping clusters of Hürthle cells with prominent nucleoli, scanty colloid and background necrosis, possibility of HCC was suggested. The patient underwent total thyroidectomy. Gross examination of the cut section of the enlarged thyroid revealed lobular tan brown tumor involving both lobes and isthmus of thyroid. Microscopic examination revealed characteristic features of HCC of thyroid gland.

### Case 2

**Figure 2:**
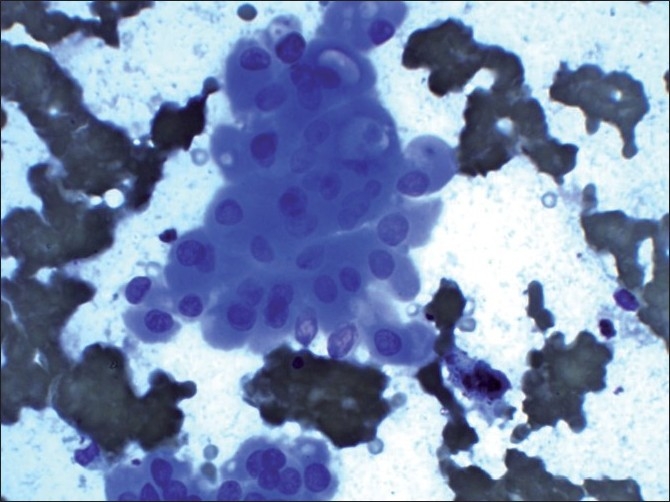
A cluster of Hürthle cells showing intracytoplasmic lumina in some cells (Giemsa, ×400)

An 83-year-old female presented with a rapidly enlarging mass in left side of neck since 6 months. The patient gave history of undergoing right hemithyroidectomy 20 years ago for multinodular goitre. On examination, a mass measuring 2.5 cm in diameter was present in the left lobe of thyroid. In addition, the cervical lymph nodes were also enlarged. FNA of the mass revealed cell-rich smears comprising Hürthle cells arranged in flat sheets, overlapping clusters and scattered singly. These cells showed pleomorphism, central to eccentrically located nucleus with single conspicuous nucleolus. Occasional cells showed intracytoplasmic lumina (ICL) [Figure 2]. Colloid was not seen in all the smears. Many bare nuclei were seen in the background. In the presence of dyscohesive as well as crowded Hürthle cells revealing pleomorphism, prominent nucleoli, intracytoplasmic lumina and absence of colloid in the background, possibility of HCC was given. The patient underwent left thyroidectomy. Histopathological examination confirmed HCC revealing capsular and vascular invasion. Cervical lymph nodes were showed metastatic tumor deposits.

## Discussion

HCC of the thyroid gland is a rare neoplasm that comprises 2–10% of all differentiated thyroid cancers.[1,2] The peak incidence occurs in the fifth to seventh decades of life. HCC usually presents as a mass in the neck, with lymphadenopathy and vocal cord paralysis.

Fine needle aspiration cytology (FNAC) is a good predictor of HCN but is of little diagnostic value in evaluating HCC, since for a tumor to be deemed malignant there has to be vascular or capsular invasion. The majority of fine-needle aspirates of the thyroid that demonstrate a predominance of Hürthle cells are diagnosed as suspicious for HCN. With a few exceptions, there has been little effort to distinguish between Hürthle cell adenomas and HCCs. As a result, in a large series, less than 10% of patients with FNA samples diagnosed as suspicious for a HCN were found to have HCC at the time of resection.[3]

There are several reasons why cytologists have not separated HCNs. First, the older literature, especially the report by Thompson *et al*.[4] suggested that all HCNs were potentially malignant and, as a result, all such tumors required resection. However, more recent, larger, and better documented series have suggested that this is not true; lesions without complete capsular penetration or vascular invasion can reliably be diagnosed as benign. Second, in a majority of studies, it has not been possible to reliably distinguish between adenomas and carcinomas.[5,6] However, in these studies, there was a strong trend to suggest that many carcinomas could be distinguished from adenomas, and the problem always was a few carcinoma cases that lacked atypia or other specific features of malignancy.[6] Finally, to our knowledge, only a few studies included more than a few cases of carcinoma, thus making it difficult to assess the value of cytological features in making this distinction.[5,6]

As the literature suggests, it is not always possible to distinguish HCCs from Hürthle cell adenomas. However, the converse is not always true; some Hürthle cell adenomas can be distinguished from HCCs. The literature and current study results clearly demonstrate that Hürthle cell adenomas have a broader range of cytological features than carcinomas. In particular, some Hürthle cell adenomas can present with an abundance of colloid. Others can have scant colloid but many Hürthle cells are found in flat, uncrowded sheets. Various studies strongly suggest that these adenomas can and should be distinguished from HCCs and should be diagnosed as benign.

Several studies have reached contradictory conclusions regarding the value of specific cytological findings in the diagnosis of HCC. Recently, a number of studies have been conducted to define criteria that are more specific for HCC, without a loss in sensitivity. Renshaw *et al*.[7] have re-examined 33 aspiration samples diagnosed as suspicious for HCN (4 non-neoplastic cases, 19 adenoma cases, and 10 carcinomas) and found that all HCCs could be identified using a total of five criteria. These criteria include: (1) predominantly Hürthle cells, (2) scant colloid, (3) at least one of either small cell dysplasia (cytoplasmic diameter less than twice the nuclear diameter, with often quite bland cells), (4) large cell dysplasia (greater than twice the variation in nuclear diameter; large cells typically demonstrate prominent nucleoli and irregular nuclear outlines), and (5) crowding (nuclei touching), and dyscohesion (single cells). Based on their results, they concluded that by focussing on criteria for HCC rather than all HCNs, criteria can be developed that improve the specificity without a loss of sensitivity. The results of the study strongly suggest that a vast majority of these conflicting data come from the fraction of Hürthle cell adenomas that have hyperplastic features (i.e., abundant colloid, flat sheets of uncrowded cells). As long as HCN is accepted as the gold standard, the data always will be confusing and contradictory.

In spite of all these criteria, in many instances, making a clear distinction between neoplastic and non-neoplastic Hürthle cell lesions may be difficult. The problems related to conventional criteria in distinguishing neoplastic and non-neoplastic Hürthle cell lesions may be due to their focus mainly on quantitative aspects of Hürthle cells, lymphocytic component, and colloid. Non-neoplastic Hürthle cell nodules in Hashimoto’s thyroiditis can yield an abundance of Hürthle cells with inconspicuous inflammation, mimicking a neoplasm. Scant or absent colloid may be seen not only in HCNs but also in Hashimoto’s thyroiditis. Although prominent nucleoli are commonly encountered in HCNs, these may also be seen in non-neoplastic Hürthle cell lesions, perhaps in response to attack by the body’s immune system.[5] Hence, there does not appear to be any single feature that is intrinsic to the neoplastic or non-neoplastic Hürthle cell lesions.

In another report, Kini identified four cytological characteristics of HCCs: (1) syncytial tumor tissue fragments, (2) small tumor cells with a high nuclear/cytoplasmic ratio, (3) prominent, regular or irregular nucleoli and (4) intranuclear cytoplasmic inclusion.[5] Using these criteria, Kini *et al*.[5] were able to identify about 60% of HCC by FNA.

Recent reports have drawn attention to two intrinsic morphological features seen in HCN, namely transgressing vessel and intracytoplasmic vacuoles.[8] González-Cámpora *et al*.[9] have shown that these vacuoles can be readily identified with electron microscope as ICL with villi and secreted material. With light microscopy, these are seen in the Diff-Quik-stained aspirates as empty vacuoles or as vacuoles with magenta material inside. The cytoplasmic vacuoles demonstrate positive immunoreaction for thyroglobulin in both aspirate smears and tissue sections. In the study conducted by Yang *et al*.,[8] transgressing vessels (TV) were identified in only neoplastic Hürthle cell lesions. Although ICL were predominantly present in true HCNs (70% of cases), the authors were able to demonstrate the presence of ICL in 15% of cases that did not represent a neoplasm. Identification of TV or ICL improved the sensitivity and specificity of cytological diagnosis of HCN over the conventional criteria. In their study, using TV or ICL as the criterion for diagnosis of HCN, they were able to obtain a sensitivity and specificity of 100 and 85%, respectively. In contrast, use of conventional criteria yielded a sensitivity and specificity of 90 and 65%, respectively. Although false-positive results may not be completely eliminated using these criteria, the improved sensitivity and specificity over conventional criteria may result in better identification of patients with HCNs compared to conventional criteria.

There is much debate regarding its clinical behavior and little is known about the long-term survival of patients with HCC. Some studies have reported a relatively benign course while others have found the tumor to behave aggressively.
